# Total flavonoids isolated from *Eucommia ulmoides* can alleviate bone loss and regulate intestinal microbiota in ovariectomized rats

**DOI:** 10.3389/fphar.2025.1513863

**Published:** 2025-02-07

**Authors:** Baocang Yin, Mingzhen Yang, Bowen Wang, Yun Zhang, Ningli Li, Qin Li, Yingying Li, Cory J. Xian, Tiejun Li, Yuankun Zhai

**Affiliations:** ^1^ The First Affiliated Hospital of Henan University, Henan University School of Stomatology, Kaifeng, China; ^2^ Kaifeng Key Laboratory of Periodontal Tissue Engineering, Kaifeng, Henan, China; ^3^ School of Pharmacy, Henan University, Kaifeng, Henan, China; ^4^ Osteoporosis Department, Luoyang Orthopedic-Traumatological Hospital, Luoyang, Henan, China; ^5^ UniSA Clinical and Health Sciences, University of South Australia, Adelaide, SA, Australia; ^6^ Department of Oral Pathology, Peking University School and Hospital of Stomatology, Beijing, China

**Keywords:** *Eucommia ulmoides*, total flavonoids, osteoporosis, Ovx, intestinal microbiota, uterus, micro-CT

## Abstract

**Ethnopharmacological relevance:**

*Eucommia ulmoides*, recognized as a traditional Chinese medicinal herb, can tonify liver and kidney and strengthen bones and muscles. Modern pharmacological research has proved that *E. ulmoides* could prohibit the occurrence of osteoporosis and arthritis.

**Aim:**

To investigate the effect and action mechanism of total flavonoids isolated from the leaves of *E. ulmoides* (TFEL) on bone loss in ovariectomized (OVX) rats, and to study its effect on intestinal flora.

**Materials and methods:**

The 3-month-old female rats were randomly divided into six groups: sham operation group, OVX model group, estradiol group, TFEL low (TFEL-L) (50), mid (-M) (100) and high (-H) (200 mg/kg/d) dose groups. After 13 weeks of treatment, the rats were sacrificed to measure bone turnover markers, related tissue biochemical indices, microstructure parameters, and osteoclastogenesis promotor RANKL and inhibitor OPG expression levels. Additionally, fecal samples were obtained for high-throughput sequencing to analyze the intestinal flora.

**Results:**

Oral administration of TFEL for 13 weeks increased the serum level of bone formation marker PINP and decreased the level of bone resorption marker NTX-I. The femoral microstructure parameters of the TFEL-M and TFEL-H groups were significantly improved compared with the OVX group, which were also confirmed by H&E histological staining. High-throughput sequencing indicated that TFEL may regulate the composition of intestinal flora and intestinal microecology.

**Conclusion:**

TFEL can prevent osteoporosis in OVX rats and has no toxic side effects. Meanwhile, TFEL can increase the diversity and improve the composition of intestinal flora in OVX rats.

## 1 Introduction

Osteoporosis (OP) is a metabolic disease characterized by bone loss and destruction of the microstructure of bone tissue (Aibar-Almazán et al., 2022). Aging and loss of sex hormones contribute to OP, especially in women who experience a sharp decline of estrogen in menopause, resulting in bone loss due to over-activated bone-resorptive cells osteoclasts and impaired bone-forming cells osteoblasts (Wu et al., 2021). Currently, approximately 200 million women globally are affected by OP, and the incidence of osteoporotic fractures is projected to double by 2050, imposing a substantial burden on healthcare systems and economies (Pisani et al., 2016). Bone metabolism is regulated by multiple factors and signaling pathways. Extensive research has demonstrated that the balance of bone metabolism is primarily governed by the Wnt signaling pathway, the OPG/RANKL/RANK signaling pathway, and the BMP-Smad signaling pathway (Cui et al., 2024; Song et al., 2022).

Pharmacotherapy is widely used in treating OP, mainly including calcitonin, bisphosphonates, vitamin D, and other medicines. While most patients can recover to a certain extent after treatment, the expensive treatment costs and significant side effects caused by long-term use make it difficult for some patients to achieve ideal results (Zhai et al., 2018). Some traditional Chinese medicines have proved to be a good choice for treating OP, because of their low costs, clinical safety, satisfactory curative effects, and long application history.

Modern pharmacological studies have demonstrated that the main active ingredients of *Eucommia ulmoides* leaves are lignans, flavonoids and iridoids, which have various effects such as lowering blood pressure, blood lipids and anti-OP (Bao et al., 2024; Deyama et al., 2001). Flavonoids are a major component of the plant, including quercetin, kaempferol, astragalin and rutin. Prior research has indicated that total flavonoids isolated from the leaves of *E. ulmoides* (TFEL) may possess the capacity to prevent and treat OP (Bao et al., 2024). In addition, TFEL can effectively inhibit the proliferation, migration, and invasion of glioblastoma cells while enhancing the body’s antioxidant capacity by increasing superoxide dismutase activity and reducing malondialdehyde levels (Bao et al., 2024; Wang et al., 2019). Furthermore, *E. ulmoides* has demonstrated the ability to prevent bone loss in rat models of retinoic acid-induced OP, with the underlying mechanism likely involving the promotion of hormone levels and the inhibition of high bone turnover rates. However, the effects and action mechanisms of TFEL in the prevention and treatment of OP caused by estradiol (E2) deficiency have been less explored.

The normal intestinal microbiota plays a regulatory role in bone mass, influencing bone metabolism by regulating the metabolic and immune status of the body, and thus maintaining bone homeostatic balance (Seely et al., 2021). In postmenopausal women, there is an abrupt and disordered change in the species ratio of intestinal flora, accompanied by an increase in immune cells and inflammatory factors, which promotes osteoclast differentiation and maturation, enhances bone resorption, and thus leads to the occurrence of OP (Xu et al., 2017). It has been suggested that improving the distribution of intestinal flora in the postmenopausal osteoporotic population may play a key role in maintaining the balance of local and systemic immune systems in the intestine and reducing bone loss (Rizzoli, 2019).

Our previous animal experiments have preliminarily clarified that TFEL can increase peak bone mass in growing rats (Zhang et al., 2024); however, whether it has a direct preventive effect on postmenopausal OP is unclear. In this study, we investigated the effects of TFEL on OP in ovariectomized (OVX) rats and its possible mechanism of action. Our findings indicated that oral administration of different doses of TFEL significantly improved bone metabolism in OVX-induced osteoporotic rats, and the mechanism may be associated with the regulation of the expression of the OPG/RANKL signaling pathway. In addition, high-throughput sequencing detection of intestinal flora revealed that TFEL could regulate intestinal flora composition, showing ameliorative effects on intestinal microecology. Data from our study suggested that *E. ulmoides* leaves have the potential to be developed into novel medicines for the prevention and treatment of postmenopausal OP.

## 2 Materials and methods

### 2.1 Isolation of total flavonoids from *Eucommia ulmoides* leaves and sources of reagents

*Eucommia ulmoides* leaves were harvested from the planting base in the School of Pharmacy, Henan University, Henan, China, and were verified by a pharmacist in the same research institute. The fresh leaves were dried at 50°C for 6 h, then crushed and screened with 24 mesh to obtain powder. The leaf powder was then soaked with 60% ethanol for 30 min at a ratio of 1:20, extracted twice (5 min each time) by using the flash extraction method. After removing the solvent by using a vacuum evaporator, ethanol extracts were obtained. For initial purification, the ethanol extracts were then subjected to macroporous resin (NKA-9) column chromatography. After removing other constituents by using H_2_O and 25% ethanol as eluent, the crude flavonoid compounds were collected using 70% ethanol as eluent, with the crude total flavonoid extract obtained after removing solvent from the eluents. The crude flavonoid extract was further purified by using macroporous resin (polyamides, 100–200 mesh) column chromatography using the same methods described to remove other ingredients and to collect the purified total flavonoids. The purity of total flavonoids was confirmed up to 85.87% by Ultraviolet spectrophotometer.

Chloral hydrate was purchased from Kemiou Chemical Reagent Company (Tianjin, China). Ethanol, xylene, neutral gum and macroporous resin (polyamides) were obtained from Sinopharm Chemical Reagent Company (Shanghai, China). The hematoxylin and eosin (H&E) staining kit (#G1003) was purchased from Servicebio Company (Wuhan, China). A kit for calcium and phosphorus analysis was supplied by Nanjing Jiancheng Bioengineering Institute (Nanjing, China). Macroporous resin (NKA-9) was purchased from Solarbio Life Sciences company (Beijing, China). Enzyme linked immunosorbent assay (ELISA) kits for the quantitative determination of bone turnover markers were purchased from Elabscience Biotechnology Company (Wuhan, China), including rat pre-collagen type I amino-terminal progenitor peptide (PINP) and rat collagen type I cross-linking amino-terminal peptide (NTX-I). Penicillin was purchased from the Hapharm Group Company (Harbin, China). MagPure Soil DNA LQ Kit was purchased from Magen Biotechnology (Guangzhou, China).

### 2.2 Experimental animal protocols

#### 2.2.1 Experimental animals, OVX and treatments

Forty-eight female Sprague-Dawley rats (3 months old of SPF grade, weighing 240 ± 10 g) were purchased from the Pengyue Experimental Animal Breeding Company (Jinan, China) and maintained at the Experimental Animal Center of School of Pharmacy at Henan University. The experimental animal qualification certificate number is SCXK (Lu) 20190003. The animals were housed and acclimatized for 7 days at 20°C ± 2°C under a 12 h light/12 h dark cycle, relative humidity of 50%–60%, and had free access to food and water. Husbandry of the animals was based on the NIH guide for the care and use of laboratory animals, and the experiment protocol was approved by the Ethics Committee of Biomedicine at Henan University. Ethics Review Committee number is HUSOM 2023-186.

All the growing rats were divided into 6 groups (n = 8 rats/group). In the sham operation (Sham) group, only a small piece of adipose tissue near the ovary was removed after opening the abdominal cavity, and the size was similar to the size of the ovarian tissue. After the animals woke up, they were kept in a single cage and heated pads were applied. Penicillin G 80,000 IU was injected daily within 3 days after operation, and at the same time, iodophor was used for disinfection to prevent infection. Ibuprofen was administered to the rats for pain management both intraoperatively and postoperatively. One week after surgery, daily intragastric administration was started. The rats in the Sham group and the model (OVX) group were given the same volume of distilled water, and the rats in estrogen (E2) group were given E2 0.208 mg/kg/d. The other three groups of rats being treated by oral gavage with different dosages of TFEL, namely, TFEL-L (low dose, 50 mg/kg body weight), TFEL-M (mid dose, 100 mg/kg), and TFEL-H (high dose, 200 mg/kg). Rats were weighed every week, and TFEL doses were adjusted accordingly. There were no deaths of rats and all rats in each group were in good mental state, so all samples were available for subsequent analysis.

#### 2.2.2 Specimen collection

After 13 weeks of treatment, all the rats were fasted overnight and were sacrificed by spinal cord dislocation after anesthetized by inhaling 2.5% isoflurane. A V-shaped incision was made in the ileocecal region by dissecting the abdomen, and the feces were collected with a sterilized toothpick into a freezing tube and stored at −80°C for 16S rRNA gene sequencing. Blood was collected from the abdominal aorta puncture and centrifuged at 2000 rpm/min for 10 min to obtain serum which was stored at −80°C until biochemical assays. The organs including the heart, liver, kidney, and uterus were removed and immediately weighed after removing their adherent connective tissues. The uterus was fixed in 10% formalin and used for histopathological examination to detect any morphological changes. Tibiae were stored frozen at −80°C and then the right femurs were collected and fixed in 75% ethanol for microcomputed tomography (micro-CT) scanning and for processing for H&E histological staining for the changes in microstructure.

### 2.3 Assays for bone turnover biomarkers in serum

Serum levels of bone formation marker PINP and bone resorption marker NTX-I were measured using ELISA kits according to the manufacturer’s instructions. Serum calcium and phosphorus levels (mmol/L) were measured using commercial kits according to the manufacturer’s instructions.

### 2.4 Micro-CT scanning and quantitative analysis

Right femurs from each group were fixed in 75% ethanol and the distal femur was scanned by an X-ray micro-CT system (SKYSCAN 1276, Bruker, Germany). Bone tissue of 2.0 mm thickness (about 100 slices) at a distance of 1 mm from the distal end of the growth plate was selected as the region of interest (ROI) for 3D reconstruction and measurements. Bone morphometric parameters including trabecular bone volume/total volume (BV/TV), trabecular thickness (Tb.Th), trabecular number (Tb.N), trabecular spacing (Tb.Sp), structure model index (SMI) and bone mineral density (BMD) were obtained by analyzing the scanned images of the ROI.

### 2.5 Sectioning and H&E staining of femurs

Femur tissue processing, sectioning and H&E staining were conducted following protocols described in a previous study with minor modification (Liu et al., 2017). After being scanned with micro-CT, the right femurs were decalcified in 14% EDTA (pH 7.5) at room temperature for 14 days with gentle rocking, with changes of fresh solution every 12 h. Subsequently, samples were washed with running tap water for 24 h and then processed by routine ethanol dehydration and paraffin embedding. Then, 5 μm longitudinal sections were cut using a Leica microtome (Leica, Germany) and placed on 5% poly-L-lysine precoated glass slides. After deparaffinization and rehydration, sections were stained with hematoxylin and then with eosin routinely, and the stained sections were scanned and analyzed with a digital slide pathological section scanner (3DHISTECH, Hungary).

### 2.6 RT-qPCR assay

The tibial bone frozen at −80°C was taken out and ground to powder in a mortar and pestle. Then 100 mg powder was placed in a homogenization tube and was fully ground in RNA extraction solution added until there was no visible tissue mass. Total RNA was extracted, and the concentration and purity of RNA were detected by Nanodrop 2000. After the total RNA was reverse-transcribed to cDNA, a fluorescent quantitative PCR reaction using the cDNA samples was performed to detect the mRNA expression of receptor activator of nuclear factor (NF)-κB-ligand (RANKL) and osteoprotegerin (OPG) in relation to the GAPDH. Servicebio Company (Wuhan, China) provided primers, the sequences of which were shown in Supplementary Table S1.

### 2.7 Western blot analysis

A 200 mg sample powder (ground during extraction of RNA) was placed in a homogenizing tube, and a lysate buffer was added to prepare protein samples. Protein concentrations were detected using a BCA kit. The protein samples were separated by SDS-PAGE gel electrophoresis and transferred to a PVDF membrane. After being blocked in skimmed milk at room temperature for 2 h, the membrane was incubated with the corresponding primary antibody (anti-RANKL, anti-OPG or anti-beta-actin) at 4°C overnight and then incubated with the corresponding secondary antibody at room temperature for 2 h. ECL luminescent reagent was added to the PVDF membrane to develop the immunoreaction bands. The optical density of the target band in relation to the internal control beta-actin band was analyzed using the Alpha software processing system.

### 2.8 Fecal flora DNA extraction and 16S rRNA sequencing

The DNA extraction kit was used to extract the DNA of the intestinal flora in the fecal samples following instructions of MagPure Soil DNA LQ Kit. The concentration of DNA was detected by agarose gel electrophoresis and NanoDrop 2000. The extracted DNA at 1 ng/μL was used as the template to perform PCR on the V3V4 region using the primers (front-end primer: 343F, TACGGRAGGCAGCAG; back-end primer: 798R, AGGGTATCTAATCCT). Amplification systems were shown in Supplementary Tables S2–S5. After amplification, the PCR products were quality-checked by 2% agarose gel electrophoresis, and the target bands were recovered by gel cutting. Finally, the PCR products were subjected to double-end sequencing using the Hiseq2500 PE250 sequencing platform.

### 2.9 Bioinformatics analysis

Illumina MiSeq sequencing was used to generate the raw double-ended sequences, which were then decontaminated using Trimmomatic software. Meanwhile, UCHIME was utilized to detect and remove chimeric sequences from the sequences. After the sequencing data were preprocessed to generate high-quality sequences, the sequences were categorized into multiple operational taxonomic units (OTUs) using Vsearch software. Representative sequences of each OTU were selected using the QIIME software package, and they were annotated against the Silva (version138) database for species comparison using the RDP classifier software. Differences and similarities in the flora of each sample were counted at phylum, class, order, family, genus, and species taxonomic levels, respectively.

Alpha diversity analysis was used to analyze the abundance of microbial communities within the samples, including the richness index (Chao1 index), the diversity index (Simpson/Shannon index), the Observed species index and the phylogenetic diversity (PD) whole tree index, and the diversity index curve was plotted. The dilution curve can directly assess the reasonableness of the amount of sequencing data and indirectly reflect the richness of species in the samples; and the flatter the curve is, the more reasonable the amount of sequencing data is.

Beta diversity analysis is mainly used to compare the differences between the microbial community structures of different samples. The evolutionary relationship and abundance information between the samples are used to calculate the distance between the samples, reflecting whether there are significant microbial community differences between the sample groups. The commonly used analysis methods, namely, the principal coordinate analysis (PCoA analysis) and non-metric multidimensional scaling analysis (NMDS analysis) were used.

### 2.10 Statistical analyses

All the data in our examinations were derived from at least three replicates, with some detections even having six duplicates. All data were presented as mean ± standard deviation (*S.D.*) and analyzed using one-way ANOVA by using SPSS 22.0 software (Chicago, IL). Pair-wise comparisons between groups were made using the Fisher’s least significant difference (LSD) method using the same software. Unifrac distances were calculated using Qiime software (version 1.9.1) for principal coordinate analysis. The statistically significant difference was considered at the level of *P*-values <0.05.

## 3 Results

### 3.1 TFEL reduced body weight in OVX rats and had no significant effect on organ index and uterine tissue morphology

**FIGURE 1:**
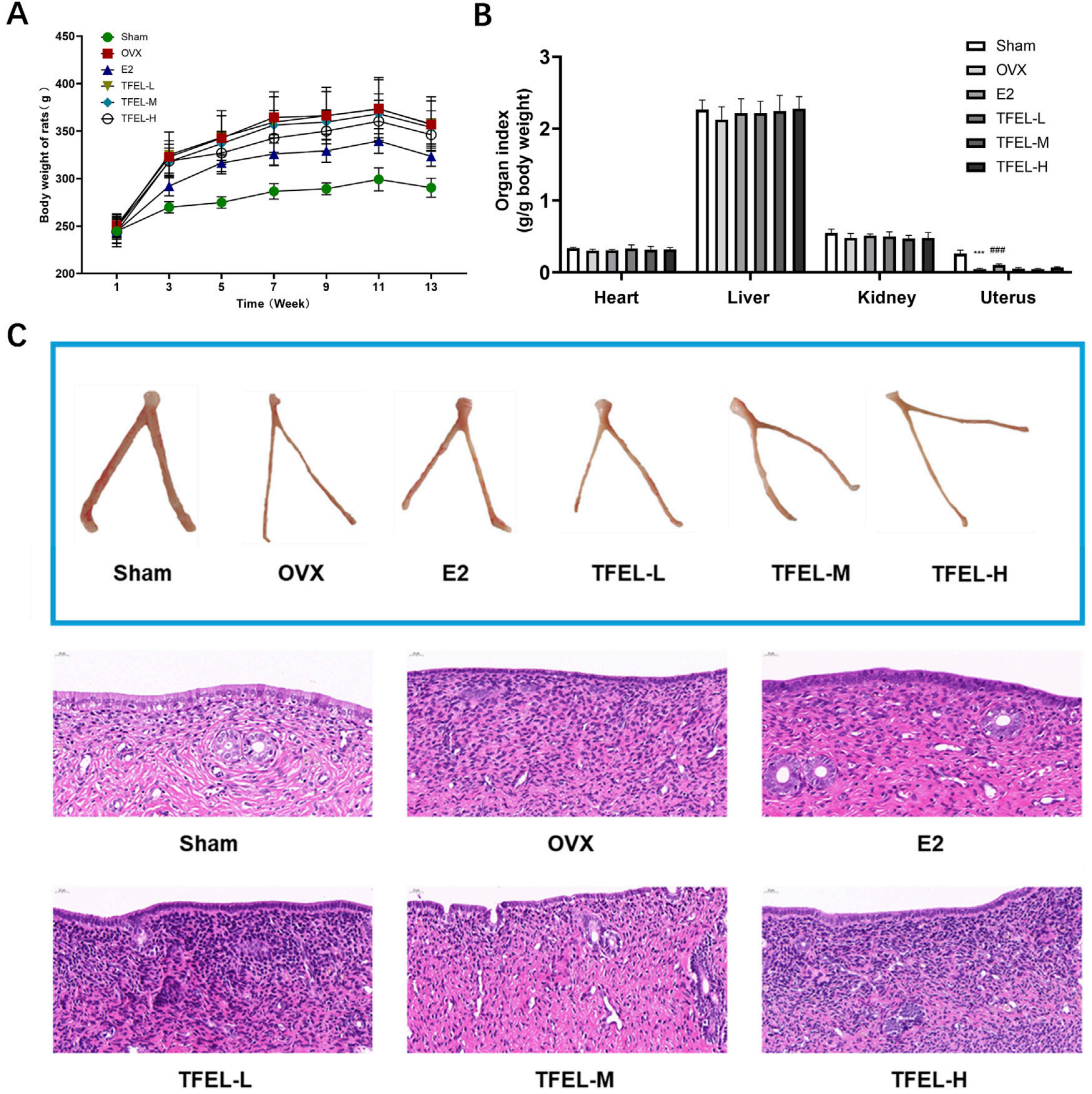
Evaluation of the total flavonoids isolated from *Eucommia ulmoides* leaves (TFEL) in OVX rats. **(A)** Changes in body weights from week 1 to the end point of the 13-week experiment of OVX rats orally gavaged with E2 (0.208 mg/kg/day) and TFEL at a low (L, 50), mid (M, 100) or high (H, 200 mg/kg/day) dose or just with water (Sham and OVX groups); **(B)** Organ index (ratio of organ weight to the total body weight) of main organs (the heart, liver, kidney and uterus); **(C)** Morphological changes of the uterus as examined by using hematoxylin/eosin ,(H&E)-stained sections. Values are means ± *S.D*., n = 8, ***P < 0.001 vs. Sham, ###*P* < 0.001 vs. OVX.

Starting from week three during the 13 weeks of administration, the body weights of rats in the OVX group were significantly higher compared to the Sham group (*P* < 0.001), and the body weights of rats in the TFEL-treated groups were all reduced to different degrees compared to the OVX group despite the lack of significant statistical differences (Figure 1A). At the end of the 13 weeks of administration, effects on wet weights of heart, kidney, liver and uterus were expressed as organ indices (organ/body weight ratios) (Figure 1B). Compared with the Sham group, the uterine index of rats in all other groups (with OVX) was significantly lower (*P* < 0.001), and there was no significant difference in other organ indices, which indicates successful rat OVX modeling. In the E2 group, the uterine index of rats was significantly higher than that of the OVX group, while the uterine index of rats in each of the TFEL-treated groups was close to that of the OVX group (Figure 1C). The uterine pathological observations further confirmed the safety of TFEL administration.

### 3.2 TFEL improved bone microstructure and enhanced bone mineral density in OVX rats

**FIGURE 2:**
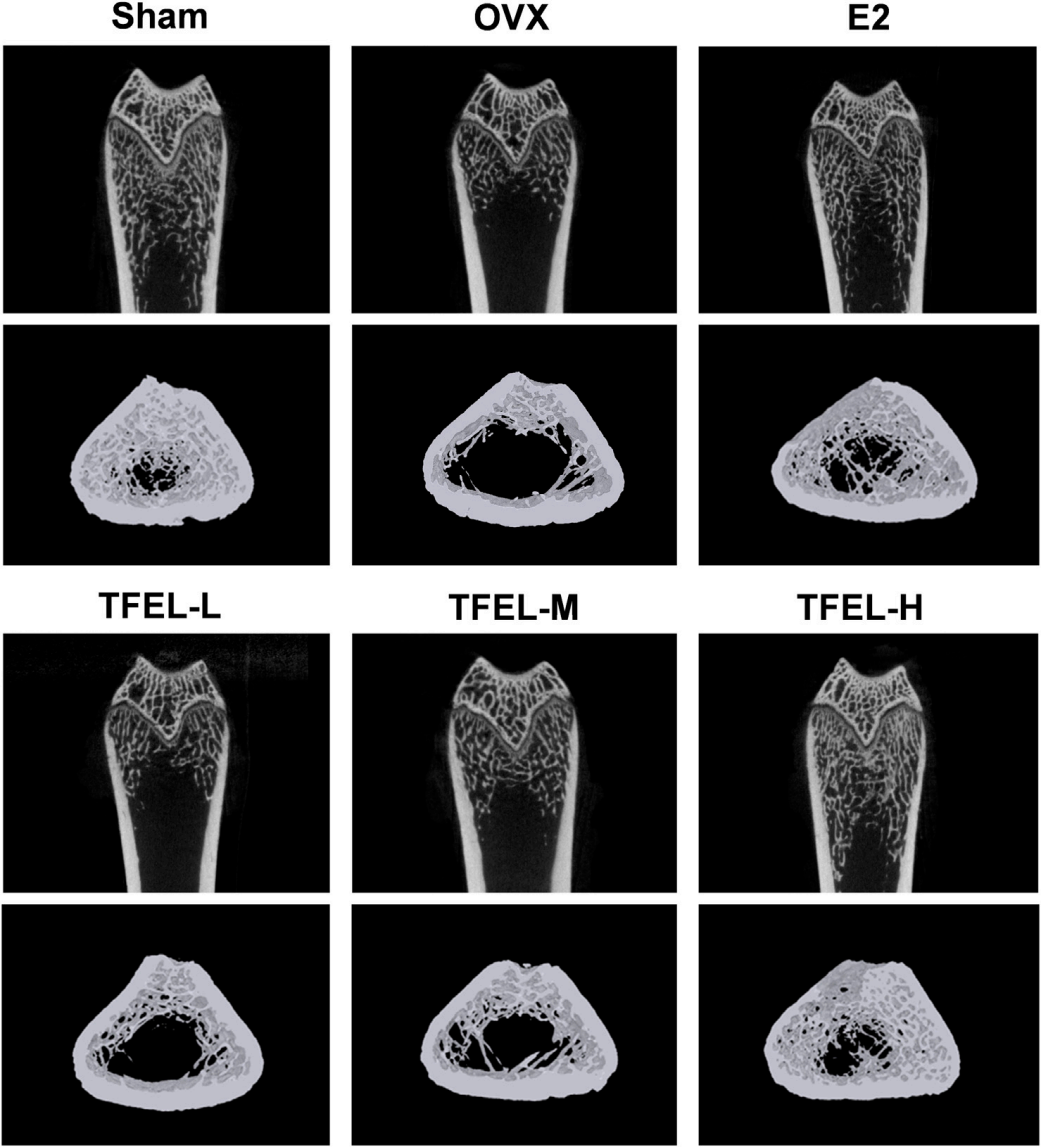
Effects on bone microstructures as examined by using micro-CT scanning of right femurs of OVX rats after being treated with E2 (0.208 mg/kg/day) and different doses of TFEL (-L, M, or -H) (50, 100, or 200 mg/kg/day) for 13 weeks or just with water (Sham and OVX groups). Representative images of the 3D architecture of trabecular bone within the distal femur metaphyseal region.

Micro-CT scanning of the distal femur examined the osteogenic effect of TFEL administration on the volume and structure of trabecular bone in the metaphysis region (Figure 2; Table 1). The trabecular bone in the Sham group was tightly aligned with little separation, whereas in the OVX group, the trabecular bone was sparsely aligned with large gaps between the trabeculae. The bone microstructural parameters were better in the E2 and the TFEL-H groups when compared with those in the OVX group, including a tightly structured trabecular bone with little separation and better continuity (Figure 2). The quantitative analysis was shown in Table 1, where bone volume over total volume fraction (BV/TV), trabecular thickness (Tb.Th), trabecular number (Tb.N) and bone mineral density (BMD) were significantly lower and trabecular spacing (Tb.Sp.) was significantly higher in the OVX group when compared with the Sham group (*P* < 0.01). However, bone microarchitecture was significantly improved after treatment with TFEL or E2, with increased levels of BV/TV, Tb.Th, Tb.N, and BMD, and decreased levels of Tb.Sp. There were significant differences between the E2, TFEL-M, and TFEL-H groups when compared with the OVX group in BV/TV, Tb.N, Tb.Sp and BMD (*P* < 0.01).

**TABLE 1:** Group comparisons of femur micro-CT scanning data.

Group	BV/TV (%)	Tb.Th (mm)	Tb.N (1/mm)	Tb.Sp (mm)	BMD (g/cm^-3^)
Sham	36.463 ± 4.039	0.127 ± 0.005	3.439 ± 0.236	0.207 ± 0.024	0.424 ± 0.037
OVX	5.800 ± 1.879***	0.101 ± 0.006**	0.762 ± 0.200***	1.384 ± 0.131***	0.130 ± 0.040***
E2	34.931 ± 4.527^###^	0.126 ± 0.004^##^	2.866 ± 0.434^###^	0.312 ± 0.051^###^	0.371 ± 0.043^###^
TFEL-L	28.820 ± 3.120^###^	0.104 ± 0.012	1.223 ± 0.297	0.848 ± 0.093^###^	0.169 ± 0.019
TFEL-M	31.966 ± 2.302^###^	0.110 ± 0.006	2.065 ± 0.394^##^	0.526 ± 0.080^###^	0.240 ± 0.029^##^
TFEL-H	34.942 ± 3.622^###^	0.113 ± 0.005	2.371 ± 0.226^###^	0.304 ± 0.023^###^	0.332 ± 0.037^###^

Sham operation (Sham) group, ovariectomized (OVX) model group, estradiol (E2, 0.208 mg/kg/d) group, and TFEL (-L, M, or -H) (50, 100, or 200 mg/kg/day) dose groups; Microstructure parameters, including bone volume over total volume (BV/TV), trabecular thickness (Tb.Th), trabecular number (Tb.N), trabecular spacing (Tb.Sp.), and bone mineral density (BMD). Values are means ± *S.D*., n = 8, **P < 0.01, ***P < 0.001 vs. Sham, ##*P* < 0.01, ###*P* < 0.001 vs. OVX.

### 3.3 TFEL promoted bone formation and reduced bone resorption in OVX rats

**FIGURE 3:**
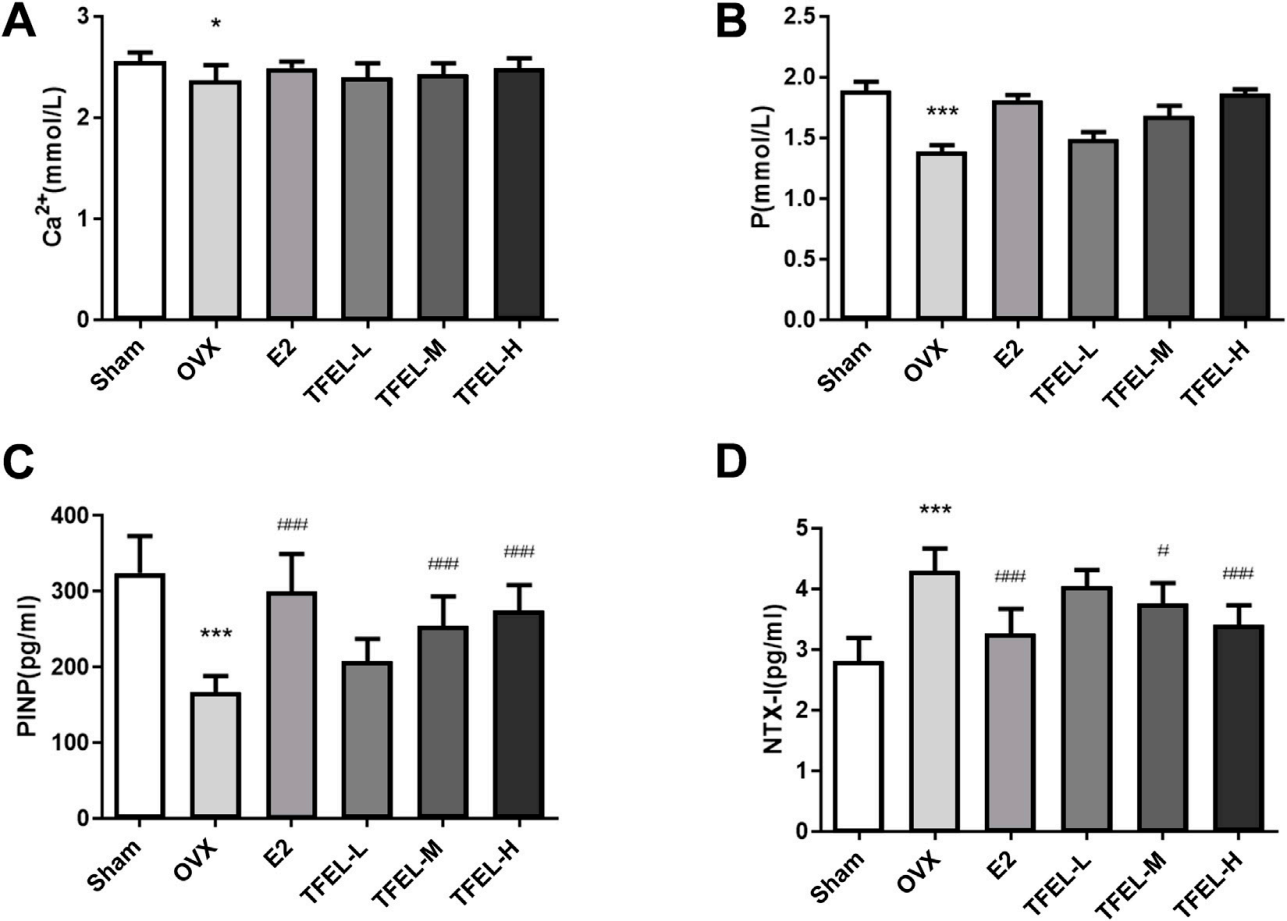
Effects on the levels of calcium, phosphorus and bone turnover markers in the serum of OVX rats after being treated with E2 (0.208 mg/kg/day) and different doses of TFEL (-L, M, or -H) (50, 100, or 200 mg/kg/day) for 13 weeks or just with water (Sham and OVX groups). **(A)** Calcium (Ca) content in serum; **(B)** Phosphorus (P) content in serum; **(C)** PINP level in serum as an indicator of bone formation; **(D)** NTX-I level in serum as an indicator of bone resorption. Values are means ± *S.D.*, n = 8, *P < 0.05, ***P < 0.001 vs. Sham, #*P* < 0.05, ###*P* < 0.001 vs. OVX.

Serum biochemical analysis results (Figures 3A, B) showed that the serum calcium and phosphorus levels of rats in the OVX group were significantly lower than those in the Sham group (*P* < 0.05), and slightly increased after TFEL treatment despite the lack of significant differences. Compared with the Sham group, the level of bone formation marker PINP was significantly lower in the OVX group (*P* < 0.001), and it was elevated significantly after treatment with E2, TFEL-M or TFEL-H when compared with the OVX group (*P* < 0.001) (Figure 3C). As shown in Figure 3D, the level of bone resorption marker NTX-I was significantly increased in the OVX group compared with the Sham group (*P* < 0.001), and it was decreased significantly after E2, TFEL-M or TFEL-H treatment when compared with the OVX group (*P* < 0.05). These data confirmed that TFEL treatment has the dual function of promoting bone formation and reducing bone resorption, thereby alleviating bone loss in OVX rats.

### 3.4 TFEL accelerated bone trabeculation and attenuated resorption in OVX rats

**FIGURE 4:**
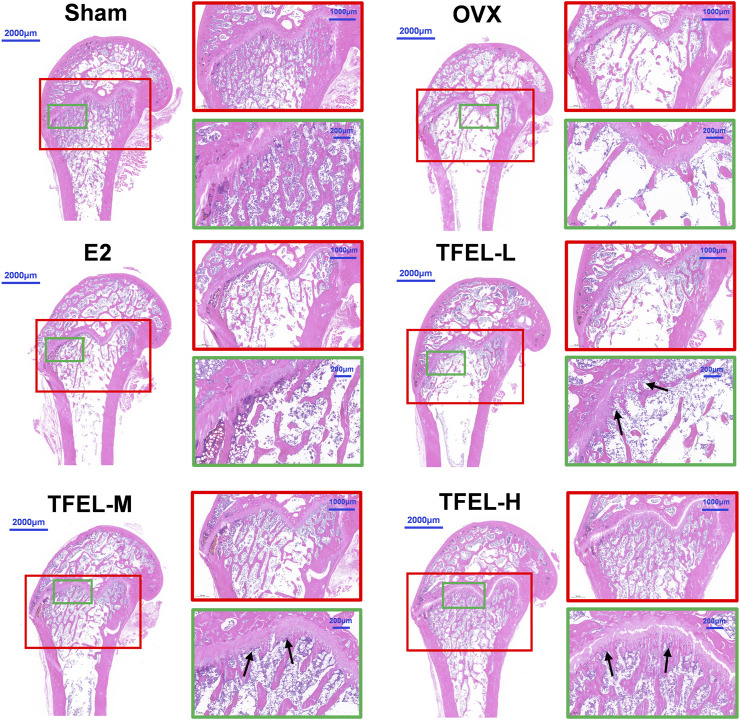
Representative images of hematoxylin/eosin (H&E)-stained sections to show the microstructure changes in the right femurs of OVX rats after being treated with E2 (0.208 mg/kg/day) and different doses of TFEL (-L, M, or -H) (50, 100, or 200 mg/kg/day) for 13 weeks or just with water (Sham and OVX groups). The red and green squares show enlarged details, and the black arrows show the changes in the osteoblasts on trabecular bone surfaces.

To further analyze TFEL treatment effects on bone, the changes in bone histomorphology in the metaphysis, growth plate, and epiphysis regions of femurs were examined in H&E-stained histology sections (Figure 4). In the Sham group, the trabeculae appeared regular and arranged neatly. In the OVX group, the density of trabeculae became smaller, the trabeculae had disordered arrangements, the bone mass was significantly reduced, and the adipocyte density in the bone marrow cavity was significantly increased, which further suggested that the rat OVX model was successfully established. Compared with the OVX group, the TFEL treatment group had thicker and more complete trabeculae and had a higher osteoblast density on the trabecular surface (black arrows in Figure 4). These results were consistent with the data from micro-CT analysis, indicating that TFEL protects the bone against trabecular bone loss or microstructure damage in OVX rats.

### 3.5 TFEL regulated the expression of osteoclastogenesis regulatory molecules OPG and RANKL

**FIGURE 5:**
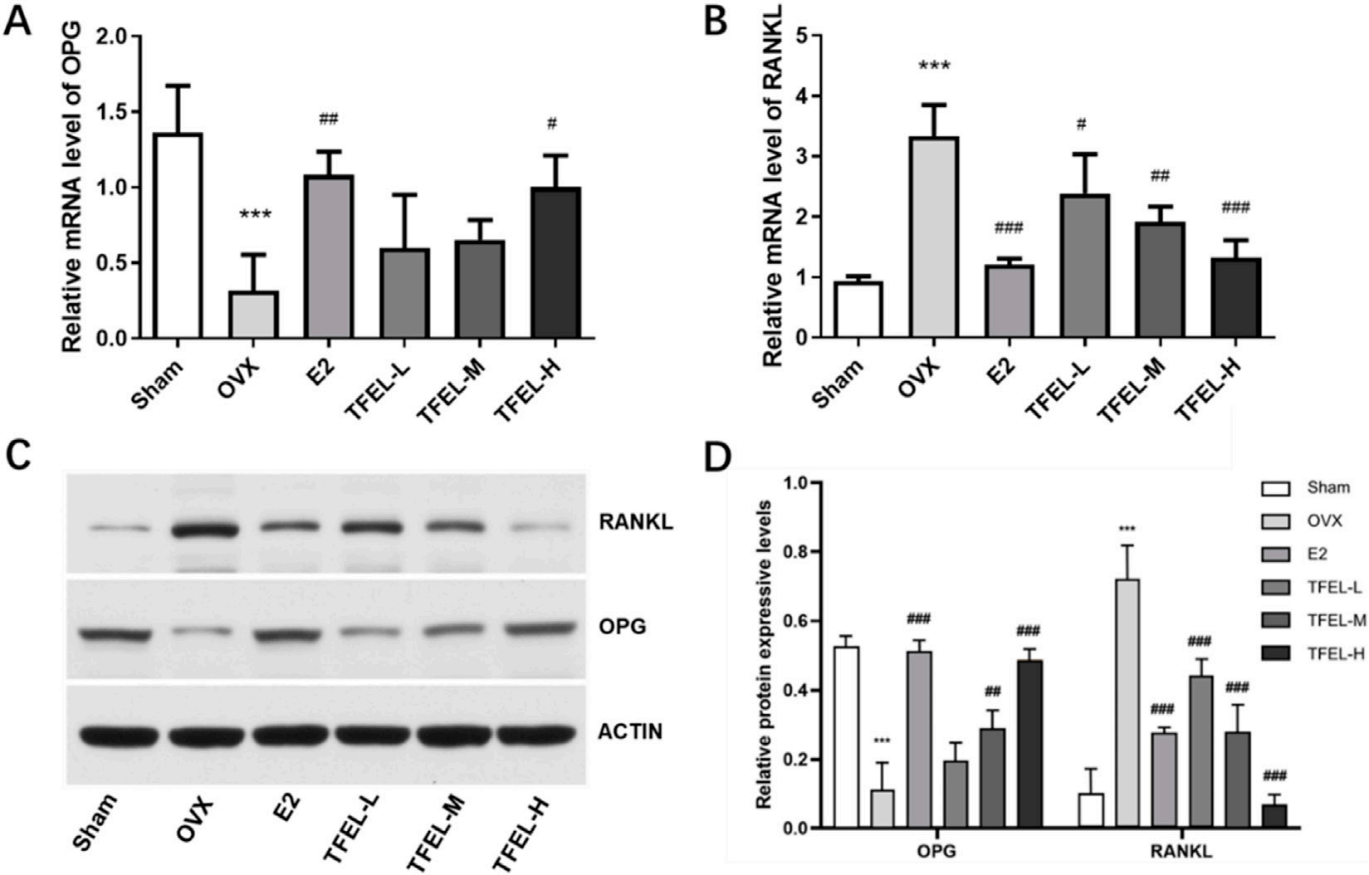
Effects on OPG and RANKL relative expression at mRNA and protein levels of OVX rats after being treated with E2 (0.208 mg/kg/day) and different doses of TFEL (-L, M, or -H) (50, 100, or 200 mg/kg/day) for 13 weeks or just with water (Sham and OVX groups). **(A)** Relative mRNA level of OPG in bone powder (relative to GAPDH internal control); **(B)** Relative mRNA level of RANKL in bone powder (relative to GAPDH internal control); **(C)** Protein expression of OPG and RANKL in bone powder; **(D)** Quantification of relative protein expression of OPG and RANKL in bone powder (relative to actin internal control) Values are means ± *S.D.*, n = 8, ***P < 0.001 vs. Sham, #*P* < 0.05, ##*P* < 0.01, ###*P* < 0.001 vs. OVX.

The OPG/RANKL signaling pathway plays a key role in regulating bone remodeling (Tyrovola, 2017). OPG, being a decoy receptor of RANKL and competitively binding to RANKL, blocks the connection pathway between RANKL and RANK, promotes osteoclast apoptosis, inhibits bone resorption, and thus regulates bone metabolism (Ostrowska et al., 2012). The mRNA and protein expression results in Figure 5 showed that the relative expression of OPG in the OVX group was significantly lower than that in the Sham group at both mRNA and protein levels, and the expression of RANKL was significantly higher than that in the Sham group (*P* < 0.001). After treatment with TFEL or E2, the expression of OPG was higher and the expression of RANKL was significantly lower than that of the OVX group (*P* < 0.05). The above data indicated that TFEL treatment can increase the OPG/RANKL ratio, reduce the binding between RANKL and RANK, and then reduce osteoclast formation and bone resorption, thereby achieving the curative effect of prevention/treatment of estrogen deficiency-induced bone loss.

### 3.6 TFEL affected the abundance of intestinal microbiota in OVX rats

**FIGURE 6:**
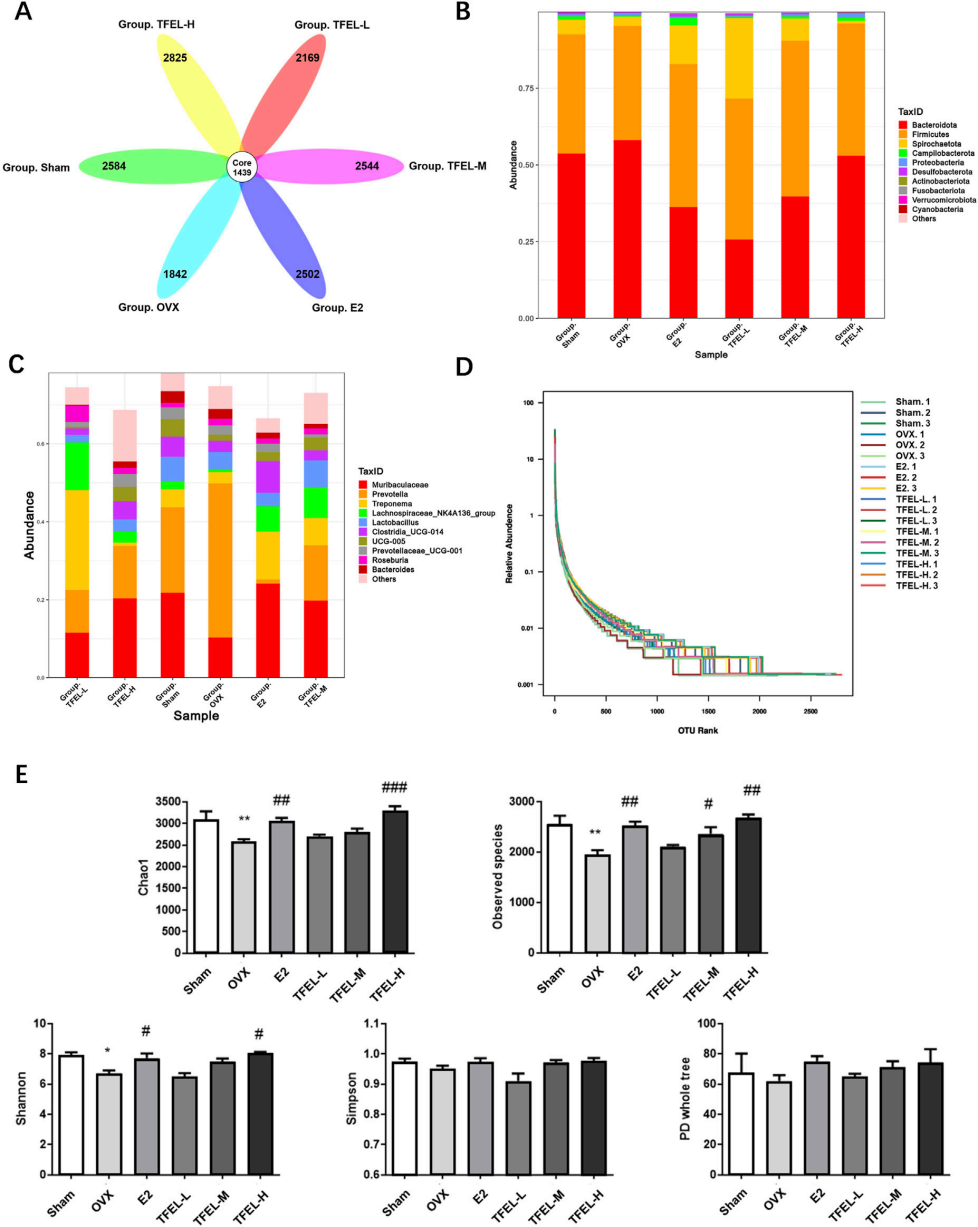
Effects on abundance and Alpha diversity analysis of the intestinal microbiota of OVX rats after being treated with E2 (0.208 mg/kg/day) and different doses of TFEL (-L, M, or -H) (50, 100, or 200 mg/kg/day) for 13 weeks or just with water (Sham and OVX groups). **(A)** Operational taxonomic unit (OUT) flower plot; **(B)** Effects on the phylum classification level of intestinal flora; **(C)** Effects on the genera classification level of intestinal flora; **(D)** Rank abundance curves; **(E)** Quantification of Alpha diversity index analysis (Chao1 index, Observed species index, Shannon index, Simpson index, and phylogenetic diversity or PD whole tree index). Values are means ± *S.D*., *P < 0.05, **P < 0.01 vs. Sham, #*P* < 0.05, ##*P* < 0.01, ###*P* < 0.001 vs. OVX.

For the fecal microbiota micro-sequencing results, the sample tag distribution table is shown in Supplementary Table S2, and the operational taxonomic unit (OUT) analysis results are shown in Figure 6A. There were 1439 OTUs in the six groups. OTUs unique to the Sham, OVX, E2, TFEL-L, TFEL-M, and TFEL-H group were 2584, 1842, 2502, 2169, 2544 and 2825, respectively. The results suggest that the abundance of fecal microbiota was reduced after OVX but was rectified after E2 or TFEL treatment when compared with that in the OVX group.

At the phylum level, *Bacteroidota* and *Firmicutes* were the absolute dominant phylum in the six groups, but there were individual differences in each sample. Compared with the OVX group, the TFEL and E2 treatment groups had varying degrees of reduction in the abundance of *Bacteroidota*, but an increase in the abundance of *Firmicutes* (*P* > 0.05) (Figure 6B). At the genus level, the relative abundance of Muribaculaceae increased in a dose-dependent manner after TFEL treatment. In contrast, the relative abundance of the *Prevotella* decreased in all treatment groups (Figure 6C).

### 3.7 Alpha diversity analysis of intestinal flora in each group of rats

The sample dilution curve and species accumulation curve, respectively, proved that the amount of sequencing data of the samples was reasonable and that the sample was sufficiently sampled for data analysis (Supplementary Figures S1, S2). The rank abundance curve showed that the OVX group generally had a smaller span on the horizontal axis than the E2 or TFEL treatment and Sham groups, indicating that the intestinal flora had changed in abundance after ovariectomy (Figure 6D). Analysis of the Alpha diversity index showed that after TFEL and E2 treatment, the diversity index was higher than that of the OVX group and that the Chao1 index, Observed species index, and Shannon index of the TFEL-H group and the E2 group were significantly higher than those of the OVX group (*P* < 0.05) (Figure 6E).

### 3.8 Beta diversity analysis of intestinal flora in each group of rats

**FIGURE 7:**
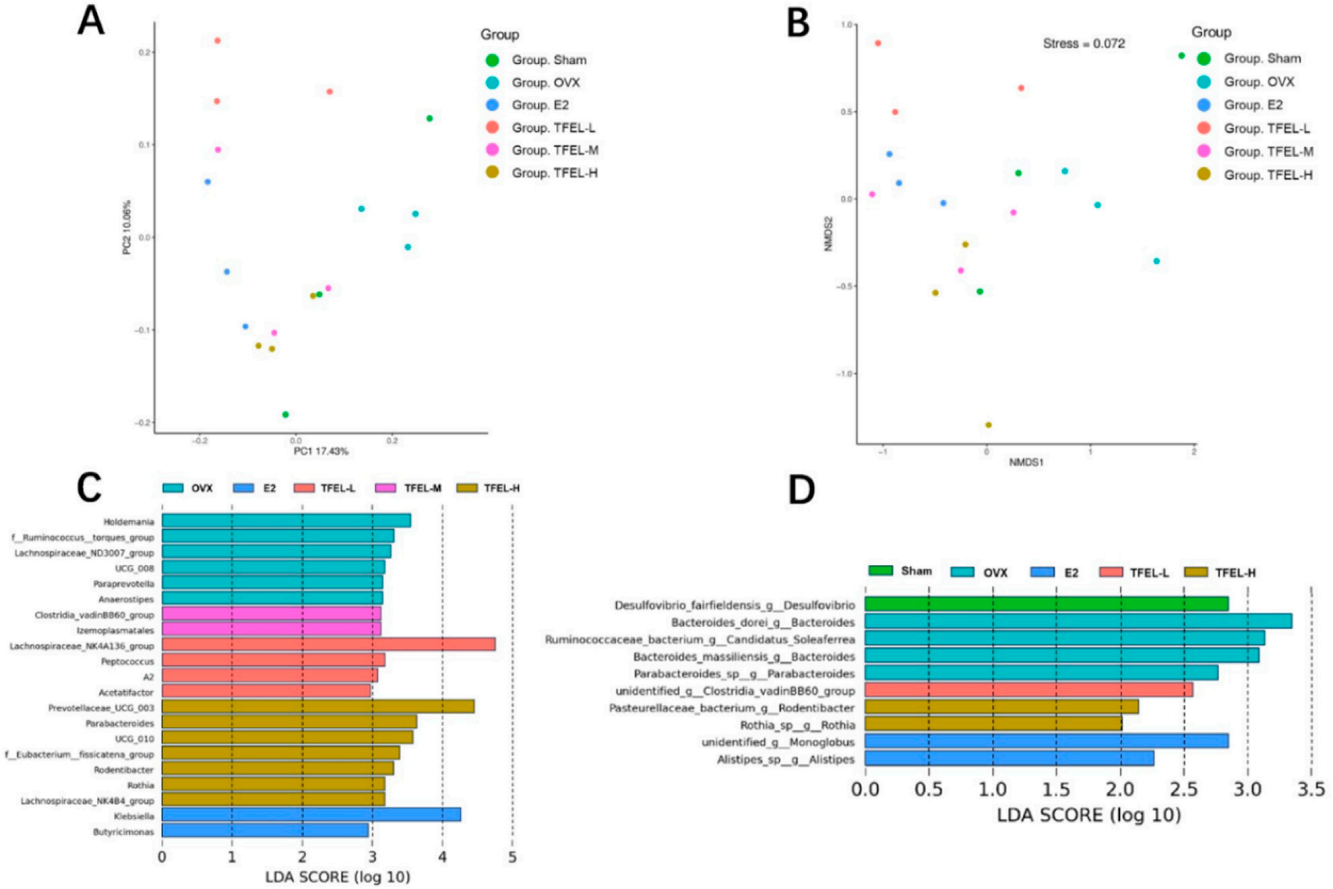
Effects on Beta diversity analysis and signature intestinal microbiota of OVX rats after being treated with E2 (0.208 mg/kg/day) and different doses of TFEL (-L, M, or -H) (50, 100, or 200 mg/kg/day) for 13 weeks or just with water (Sham and OVX groups). **(A)** The principal coordinate analysis (PCoA analysis, 2D); **(B)** The non-metric multidimensional scaling analysis (NMDS analysis, 2D); **(C)** Linear discriminant analysis effect size (LDA Effect Size) analysis of signature intestinal microorganisms in genus level; **(D)** LDA Effect Size analysis of signature intestinal microorganisms in species level.

Results of the principal coordinate analysis (PCoA) based on the unweighted-unifrac distance (Figure 7A) showed that the Sham group and the OVX group had different aggregation tendencies, indicating differences in the structural composition of the intestinal microbiota between the two groups. The points in the TFEL-H group were more concentrated and were close to the Sham group on the PC1 axis. Results of the non-metric multidimensional scaling analysis (NMDS) based on unweighted-unifrac distance (Figure 7B) showed that three samples from the OVX group were clustered together, which was a significant difference compared to the Sham group (the distance between the dots represents the degree of difference in the flora structure). After E2 or TFEL treatment, the samples from each treatment group were all clustered together except for a few samples that deviated from the whole, indicating that there were differences in the composition of intestinal flora between the treatment groups and the OVX group.

### 3.9 Effects of TFEL on the signature intestinal microbiota of OVX rats

To explore the effect of TFEL intervention on the intestinal flora even further, linear discriminant analysis effect size (LDA Effect Size) analysis was used to assess the species of the flora from phylum to genus in each group. According to the analysis results at the genus level, the intestinal flora of rats in the OVX, E2, TFEL-L, TFEL-M, and TFEL-H groups were significantly enriched with 6, 2, 4, 2, and 7 signature intestinal microbial species, respectively (Figure 7C). Signature species were also obtained based on the analysis results at the species level, with the Sham, OVX, E2, TFEL-L, and TFEL-H groups being mainly enriched with 1, 4, 2, 1, and 2 species, respectively (Figure 7D).

## 4 Discussion

Osteoporosis (OP) is a metabolic bone disease caused by many factors, among which postmenopausal OP caused by E2 deficiency is the most common type of OP (Eastell and Szulc, 2017). Due to the decline of ovarian function in postmenopausal women, the level of E2 secreted by the ovary decreases, resulting in this bone metabolism disorder, bone mass reduction, and bone density reduction (Diab and Watts, 2013). For postmenopausal OP, menopausal estrogen plus progestogen therapy has shown a certain efficacy; however, long-term usage of the hormone replacement therapy increases the risk of cardiovascular disease and breast cancer (Rossouw et al., 2002). Thus, there is still a requirement for newer, safer, and more effective therapeutic options or agents for OP therapy and prevention. In recent years, traditional Chinese medicine has attracted worldwide attention because of its therapeutic effects for OP, lower adverse effects, higher clinical safety, and long-term application history.

*Eucommia ulmoides Oliv.* has been used in traditional Chinese medicine formulas for nearly 2,000 years and is known to tonify the liver and kidneys and strengthen the muscles and bones (Huang et al., 2021). Flavonoids are a large family of plant metabolites that have multiple pharmacological effects, and 13 flavonoids have been obtained and identified from the barks, leaves and seeds of *E. ulmoides*, including kaempferol, astragalin, quercetin, isoquercetin, rutin, hirsutin, and wogonside (He et al., 2014). Our previous animal studies have initially confirmed that TFEL can increase the peak bone mass of growing rats (Zhang et al., 2024), however, whether TFEL has a direct preventive effect on postmenopausal OP is not clear. Therefore, this study addressed this issue by oral administration of TFEL for 13 weeks in OVX rats.

Research has demonstrated that the alcohol extract from *E. ulmoides* leaves can regulate bone metabolic balance, elevate serum estradiol levels, and increase bone mineral density in OVX osteoporotic rats (Liu et al., 2018). Additionally, studies have indicated that flavonoids derived from *E. ulmoides* leaves significantly inhibit the proliferation of MG-63 osteoblasts, which is likely associated with the activation of TLR-4, MyD88, and NF-κB signaling pathways (He and Ti, 2021). In this study, the pathologic section of the femur of rats in the OVX group showed disordered arrangement of bone trabeculae, obvious reduction of bone volume, and obvious increase of adipocytes in the bone marrow cavity, indicating that the rat OVX OP model was successfully established. After TFEL treatment, the levels of OP markers BV/TV, BMD, Tb.Th and Tb.N were increased, and the level of Tb.Sp was decreased, with the number of bone trabeculae in rats being significantly increased and more tightly connected. Consistently, the level of serum bone resorption marker NTX-I was decreased, and the level of bone formation marker PINP was increased with the TFEL or E2 treatment. These indicated that TFEL could increase bone density, improve the OP status of OVX rats and alleviate bone loss. Furthermore, the long-term (13 weeks) TFEL oral administration also alleviated E2 deficiency-induced weight increase and had no proliferative or stimulatory effects on the uterus and other organs, which strongly suggested that TFEL can be used as a food additive for daily life or other uses to improve bone health.

Since the OPG/RANKL pathway is key to regulating bone metabolism, and the expression ratio of OPG/RANKL is an important indicator for evaluating bone homeostasis (Chen et al., 2018), we detected the relative expression of OPG and RANKL in each group to explore whether the mechanism of TFEL bone protective effect was related to this pathway. The results showed that TFEL upregulated the relative expression of OPG and downregulated the relative expression of RANKL in OVX rats at both the mRNA and protein levels, increasing the OPG/RANKL ratio and a decrease in bone resorption, thus achieving the efficacy of TFEL in preventing E2 deficiency-induced bone loss in OVX rats, which may be one of the important mechanisms by which TFEL ameliorates postmenopausal OP.

In recent years, some studies have shown that intestinal flora is closely related to the regulation of bone metabolism in the body. Intestinal flora is the largest microbial system in the human body, which is closely related to human health, and its number is dozens of times that of human cells. Intestinal flora can regulate bone metabolism through a variety of pathways, mainly including calcium and phosphate absorption, immunomodulation and other aspects (Shin et al., 2019). Some studies have found that germ-free mice, compared to normal mice, have higher bone mass, bone mineral density and bone volume fraction, and lower levels of bone resorption markers, suggesting that intestinal flora have a regulatory role in bone metabolism (Ohlsson and Sjögren, 2015; Yan and Charles, 2017). To investigate whether modulating the intestinal microflora is an important target or one mechanism of TFEL action for OP treatment, we used 16S rRNA high-throughput sequencing technology to sequence the intestinal flora of six groups of rats and analyze the changes in microbial diversity.

Our results revealed that the OVX group had the lowest number of OUTs, while the highest number of OUTs was found in the TFEL-H group. OVX rats treated with different concentrations of TFEL showed an increase in Chao1 index, Observed species index, Shannon index, Simpson index, and PD whole tree index in all the groups, which indicated that TFEL could improve the intestinal flora richness, diversity, and evenness. In addition, the analysis of the flora structure revealed that the abundance of *Bacteroidota* increased and the abundance of *Firmicutes* decreased in the OVX group compared with the Sham group, and the abundance of *Ruminococcus* and *Prevotella* both increased. This was in agreement with studies by others (Fuhrman et al., 2014; Ma et al., 2020). *Firmicutes* have immunomodulatory effects, promoting the formation of regulatory Treg cells and maintaining immune system homeostasis, and they also inhibit osteoclast differentiation and affect osteoblast formation (Atarashi et al., 2014). *Ruminococcus*, *Clostridia* and *Enterococcus* were positively correlated with osteoclastic indices and consistent with the trend of bone loss in denuded rats (Ma et al., 2020). Furthermore, OVX rats in our study showed a decrease in *Bacteroidota* abundance, an increase in *Firmicutes* abundance, and an increase in bone mass after TFEL intervention, suggesting that TFEL can ameliorate OP through intestinal flora in the postmenopausal period, but the exact mechanism is not clear.

In summary, this study preliminarily investigated the preventive and curative effects of TFEL on OP in OVX model rats, as well as its effects on the diversity of intestinal flora and the structure of the flora. It was found that oral administration of different doses of TFEL could significantly improve the level of bone metabolism in OVX rats, and the mechanism may be related to the regulation of the OPG/RANKL signaling pathway. Furthermore, TFEL could regulate the composition of intestinal flora and intestinal microecology in OVX rats, which could ameliorate postmenopausal OP. This provides a new idea for the clinical treatment of OP by regulating intestinal flora. Moreover, since *E. ulmoides* leaves have been listed as new basic food sources instead of herb sources in China since 2018 (Zhu and Sun, 2018), and TFEL is rich with various natural bioactive ingredients that have multiple functions for health, especially the positive effects on bone health, it can be developed into a superfood or utilized as a human health additive or even as a nutrition tea, all of which can help strength the bone health.

However, the sample size in this study is limited, and further molecular-level experiments are required to elucidate the underlying mechanisms of TFEL. Additionally, while this study has demonstrated that TFEL can modulate the microbiota to alleviate OP, more comprehensive investigations, including fecal microbiota transplantation and analysis of intestinal microbial metabolites, are necessary to fully uncover the regulatory mechanisms of TFEL on gut flora. Finally, further research and development are needed to make TFEL a therapy to prevent/treat postmenopausal OP, food additives, or other products to promote bone health.

## 5 Conclusion

TFEL can prevent the weight gain, bone loss, and bone microstructural degeneration caused by E2 deficiency, effectively improve the microstructure of trabeculae, and increase bone mass in OVX rats, but has no proliferative and stimulating effect on the uterus and other organs. In addition, oral consumption of TFEL can increase the diversity of intestinal flora and improve the composition of intestinal flora in OVX rats, which provides a new idea for the prevention and treatment of postmenopausal OP.

## Data Availability

The original contributions presented in the study are publicly available. This data can be found here: https://www.ncbi.nlm.nih.gov/bioproject/PRJNA1217576.

## Abbreviations

TFEL, Total flavonoids isolated from the leaves of *Eucommia ulmoides*; OVX, Ovariectomized; OP, Osteoporosis; E2, Estradiol; BV/TV, Bone volume over total volume; Tb.Th, Trabecular thickness; Tb.N, Trabecular number; Tb.Sp, Trabecular spacing; BMD, Bone Mineral Density; PINP, Pre-collagen type I amino-terminal progenitor peptide; NTX-I, Collagen type I cross-linking amino-terminal peptide; OPG, Osteoprotegerin; RANKL, Receptor activator of nuclear factor-κB-ligand; OUT, Operational taxonomic unit; PD, Phylogenetic diversity; LSD, Least significant difference.

## References

[B1] Aibar-Almazán A. Voltes-Martínez A. Castellote-Caballero Y. Afanador-Restrepo D. F. Carcelén-Fraile M. D. C. López-Ruiz E. (2022). Current status of the diagnosis and management of osteoporosis. Int. J. Mol. Sci. 23 (16), 9465. 10.3390/ijms23169465 36012730 PMC9408932

[B2] Atarashi K. Tanoue T. Oshima K. Suda W. Honda K. Nishikawa H. (2014). Treg induction by a rationally selected mixture of Clostridia strains from the human microbiota. Nature 500 (7461), 232–236. 10.1038/nature12331 23842501

[B3] Bao L. Sun Y. Wang J. Li W. Liu J. Li T. (2024). A review of “plant gold” Eucommia ulmoides Oliv.: a medicinal and food homologous plant with economic value and prospect. Heliyon 10 (2), e24851. 10.1016/j.heliyon.2024.e24851 38312592 PMC10834829

[B4] Chen X. Wang Z. Duan N. Zhu G. Schwarz E. M. Xie C. (2018). Osteoblast-osteoclast interactions. Connect. Tissue Res. 59 (2), 99–107. 10.1080/03008207.2017.1290085 28324674 PMC5612831

[B5] Cui Y. Lv B. Li Z. Ma C. Gui Z. Geng Y. (2024). Bone-targeted biomimetic nanogels Re-establish osteoblast/osteoclast balance to treat postmenopausal osteoporosis. Small Weinheim Der Bergstrasse, Ger. 20 (6), e2303494. 10.1002/smll.202303494 37794621

[B6] Deyama T. Nishibe S. Nakazawa Y. (2001). Constituents and pharmacological effects of Eucommia and Siberian ginseng. Acta Pharmacol. Sin. 22 (12), 1057–1070. 11749801

[B7] Diab D. L. Watts N. B. (2013). Postmenopausal osteoporosis. Curr. Opin. Endocrinol. Diabetes, Obes. 20 (6), 501–509. 10.1097/01.med.0000436194.10599.94 24150190

[B8] Eastell R. Szulc P. (2017). Use of bone turnover markers in postmenopausal osteoporosis. Lancet. Diabetes and Endocrinol. 5 (11), 908–923. 10.1016/S2213-8587(17)30184-5 28689768

[B9] Fuhrman B. J. Feigelson H. S. Flores R. Gail M. H. Xu X. Ravel J. (2014). Associations of the fecal microbiome with urinary estrogens and estrogen metabolites in postmenopausal women. J. Clin. Endocrinol. Metabolism 99 (12), 4632–4640. 10.1210/jc.2014-2222 PMC4255131 25211668

[B10] He X. Wang J. Li M. Hao D. Yang Y. Zhang C. (2014). Eucommia ulmoides Oliv.: ethnopharmacology, phytochemistry and pharmacology of an important traditional Chinese medicine. J. Ethnopharmacol. 151 (1), 78–92. 10.1016/j.jep.2013.11.023 24296089

[B11] He Z. Ti H. (2021). The inhibitory effect of Eucommia ulmoides leaf flavonoids on the proliferation of osteoblasts MG-63 and its mechanism. Chin. J. Pharmacol. Toxicol. 35 (10), 742–743.

[B12] Huang L. Lyu Q. Zheng W. Yang Q. Cao G. (2021). Traditional application and modern pharmacological research of Eucommia ulmoides Oliv. Chin. Med. 16 (1), 73. 10.1186/s13020-021-00482-7 34362420 PMC8349065

[B13] Liu H. Zhu R. Liu C. Ma R. Wang L. Chen B. (2017). Evaluation of decalcification techniques for rat femurs using HE and immunohistochemical staining. BioMed Res. Int. 2017, 9050754. 10.1155/2017/9050754 28246608 PMC5299168

[B14] Liu Y. Zhang B. Li W. Hu L. (2018). Effects of Eucommia leaf alcohol extract on bone metabolism biochemical indexes, bone mineral density, lL-6 and TNF-α in ovariectomized rats. Acta Chin. Med. 33 (3), 445–448. 10.16368/j.issn.1674-8999.2018.03.107

[B15] Ma S. Qin J. Hao Y. Shi Y. Fu L. (2020). Structural and functional changes of gut microbiota in ovariectomized rats and their correlations with altered bone mass. Aging 12 (11), 10736–10753. 10.18632/aging.103290 32484785 PMC7346027

[B16] Ohlsson C. Sjögren K. (2015). Effects of the gut microbiota on bone mass. Trends Endocrinol. Metabolism TEM 26 (2), 69–74. 10.1016/j.tem.2014.11.004 25497348

[B17] Ostrowska Z. Ziora K. Oświęcimska J. Swiętochowska E. Szapska B. Wołkowska-Pokrywa K. (2012). RANKL/RANK/OPG system and bone status in females with anorexia nervosa. Bone 50 (1), 156–160. 10.1016/j.bone.2011.09.054 22001124

[B18] Pisani P. Renna M. D. Conversano F. Casciaro E. Di Paola M. Quarta E. (2016). Major osteoporotic fragility fractures: risk factor updates and societal impact. World J. Orthop. 7 (3), 171–181. 10.5312/wjo.v7.i3.171 27004165 PMC4794536

[B19] Rizzoli R. (2019). Nutritional influence on bone: role of gut microbiota. Aging Clin. Exp. Res. 31 (6), 743–751. 10.1007/s40520-019-01131-8 30710248

[B20] Rossouw J. E. Anderson G. L. Prentice R. L. LaCroix A. Z. Kooperberg C. Stefanick M. L. (2002). Risks and benefits of estrogen plus progestin in healthy postmenopausal women: principal results from the Women’s Health Initiative randomized controlled trial. JAMA 288 (3), 321–333. 10.1001/jama.288.3.321 12117397

[B21] Seely K. D. Kotelko C. A. Douglas H. Bealer B. Brooks A. E. (2021). The human gut microbiota: a key mediator of osteoporosis and osteogenesis. Int. J. Mol. Sci. 22 (17), 9452. 10.3390/ijms22179452 34502371 PMC8431678

[B22] Shin H. E. Kwak S. E. Lee J.-H. Zhang D. Bae J. H. Song W. (2019). Exercise, the gut microbiome, and frailty. Ann. Geriatric Med. Res. 23 (3), 105–114. 10.4235/agmr.19.0014 PMC7370771 32743298

[B23] Song S. Guo Y. Yang Y. Fu D. (2022). Advances in pathogenesis and therapeutic strategies for osteoporosis. Pharmacol. and Ther. 237, 108168. 10.1016/j.pharmthera.2022.108168 35283172

[B24] Tyrovola J. B. (2017). The “mechanostat” principle and the osteoprotegerin-OPG/RANKL/RANK system PART II. The role of the hypothalamic-pituitary Axis. J. Cell. Biochem. 118 (5), 962–966. 10.1002/jcb.25793 27862210

[B25] Wang Y. Tan X. Li S. Yang S. (2019). The total flavonoid of Eucommia ulmoides sensitizes human glioblastoma cells to radiotherapy via HIF-α/MMP-2 pathway and activates intrinsic apoptosis pathway. OncoTargets Ther. 12, 5515–5524. 10.2147/OTT.S210497 PMC6633463 31371989

[B26] Wu D. Cline-Smith A. Shashkova E. Perla A. Katyal A. Aurora R. (2021). T-cell mediated inflammation in postmenopausal osteoporosis. Front. Immunol. 12, 687551. 10.3389/fimmu.2021.687551 34276675 PMC8278518

[B27] Xu X. Jia X. Mo L. Liu C. Zheng L. Yuan Q. (2017). Intestinal microbiota: a potential target for the treatment of postmenopausal osteoporosis. Bone Res. 5, 17046. 10.1038/boneres.2017.46 28983411 PMC5627629

[B28] Yan J. Charles J. F. (2017). Gut microbiome and bone: to build, destroy, or both? Curr. Osteoporos. Rep. 15 (4), 376–384. 10.1007/s11914-017-0382-z 28620867 PMC5538387

[B29] Zhai Y. Wang Q. Li Y. Cui J. Feng K. Kong X. (2018). The higher osteoprotective activity of psoralidin *in vivo* than coumestrol is attributed by its presence of an isopentenyl group and through activated PI3K/Akt axis. Biomed. and Pharmacother. = Biomedecine and Pharmacother. 102, 1015–1024. 10.1016/j.biopha.2018.03.166 29710518

[B30] Zhang Y. Yang M. Li N. Li Q. Li Y. Zhai Y. (2024). Total flavonoids isolated from the leaves of Eucommia ulmoides augment peak bone mass in female rats and show no side effects in other organs. Curr. Pharm. Des. 30, 2410–2423. 10.2174/0113816128298755240613100018 38963117

[B31] Zhu M.-Q. Sun R.-C. (2018). Eucommia ulmoides oliver: a potential feedstock for bioactive products. J. Agric. Food Chem. 66 (22), 5433–5438. 10.1021/acs.jafc.8b01312 29745662

