# Supplementary material for: Total flavonoids isolated from Eucommia ulmoides can alleviate bone loss and regulate intestinal microbiota in ovariectomized rats
Source: Front Pharmacol. 2025 Feb 7;16:1513863. doi: 10.3389/fphar.2025.1513863 (PMC11842935; doi:10.3389/fphar.2025.1513863)
Supplement: Supplementary file 1 [file DataSheet1.docx]

**Supplemental data**

The supplemental materials in the paper are as follows:

**Table S1** **Primer sequence** **of RT-qPCR**

| Gene | Primer sequence (5'-3') | fragment length（bp） |
| --- | --- | --- |
| OPG | Upstream: GTGGAATAGATGTCACCCTGTGC | 165 |
|  | Downstream: TTTGCTCTTGCGAGCTGTGT |  |
| RANKL | Upstream: CATCGGGTTCCCATAAAGTCAGT | 137 |
|  | Downstream: GCAAATGTTGGCGTACAGGTAAT |  |
| GAPDH | Upstream: CTGGAGAAACCTGCCAAGTATG | 138 |
|  | Downstream: GGTGGAAGAATGGGAGTTGCT |  |

**Table S2 The first step of PCR amplification reaction**

| Name | Volume |
| --- | --- |
| 2×Gflex PCR Buffer | 15μL |
| 5 pmol/μl primer F | 1μL |
| 5 pmol/μl primer R | 1μL |
| Template DNA | ≥ 1μL (50 ng) |
| Tks Gflex DNA Polymerase (1.25U/μL) | 0.6μL |
| H2O | A 30μL supplement was made |
| Total amount | 30μL |

**Table S3 The first step of PCR amplification reaction program**

| Temperature | Time | Number of cycles |
| --- | --- | --- |
| 94℃ | 5min | 1 |
| 94℃ | 30s | 26 |
| 56℃ | 30s |  |
| 72℃ | 20s |  |
| 72℃ | 5min | 1 |
| 4℃ | hold | - |

**Table S4 The second step of PCR amplification reaction**

| Name | Volume |
| --- | --- |
| 2×Gflex PCR Buffer | 15μL |
| Tks Gflex DNA Polymerase (1.25U/μL) | 0.6μL |
| Adapter I5 | 1μL |
| Adapter I7 | 1μL |
| H2O | A 30μL supplement was made |
| Total amount | 30μL |
| First step product | Take 50ng |

**Table S5 The second step of PCR amplification reaction program**

| Temperature | Time | Number of cycles |
| --- | --- | --- |
| 94℃ | 5min | 1 |
| 94℃ | 30s | 7 |
| 56℃ | 30s |  |
| 72℃ | 20s |  |
| 72℃ | 5min | 1 |
| 4℃ | hold | - |

**Table S6** **Sample tags distribution table**

| Sample ID | Clean tags | Valid tags | Valid percent | valid mean Length | OUT counts |
| --- | --- | --- | --- | --- | --- |
| Sham.1 | 75114 | 68913 | 91.74% | 420.2 | 1789 |
| Sham.2 | 76778 | 67032 | 87.31% | 413.99 | 2442 |
| Sham.3 | 77004 | 64752 | 84.09% | 411.57 | 2743 |
| OVX.1 | 74631 | 64991 | 87.08% | 413.63 | 2077 |
| OVX.2 | 77021 | 66361 | 86.16% | 416.3 | 1798 |
| OVX.3 | 76749 | 67918 | 88.49% | 416.36 | 2061 |
| E2.1 | 74341 | 65097 | 87.57% | 412.21 | 2692 |
| E2.2 | 74376 | 63873 | 85.88% | 413.23 | 2411 |
| E2.3 | 76689 | 67169 | 87.59% | 414.35 | 2548 |
| TFEL-L.1 | 75909 | 67277 | 88.63% | 414.14 | 2197 |
| TFEL-L.2 | 74573 | 65504 | 87.84% | 413.97 | 2089 |
| TFEL-L.3 | 77125 | 68389 | 88.67% | 414.13 | 2176 |
| TFEL-M.1 | 75333 | 65017 | 86.31% | 415.48 | 2343 |
| TFEL-M.2 | 75403 | 64368 | 85.37% | 410.38 | 2170 |
| TFEL-M.3 | 75046 | 65273 | 86.98% | 414.67 | 2634 |
| TFEL-H.1 | 75482 | 67485 | 89.41% | 417.12 | 2083 |
| TFEL-H.2 | 77055 | 67212 | 87.23% | 410.42 | 2683 |
| TFEL-H.3 | 76951 | 67382 | 87.56% | 412.77 | 2798 |


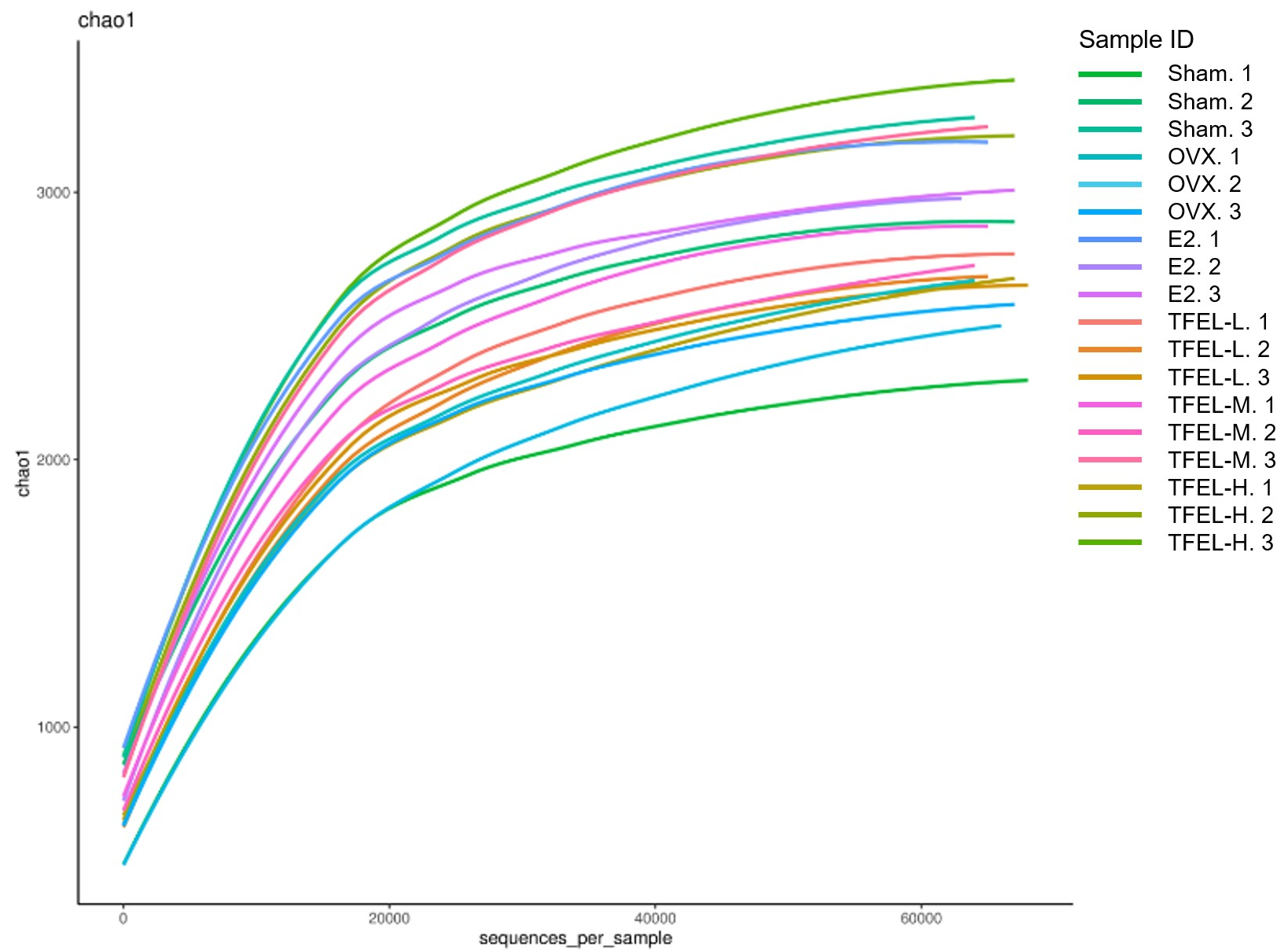


**Figure S1 Dilution curve**


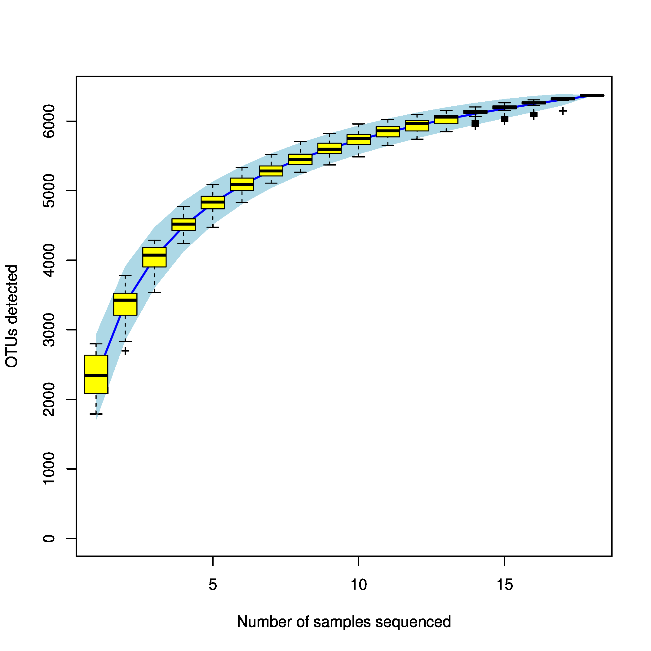


**Figure S2** **Cumulative curve of species**
